# Nonurgent patients in emergency departments: rational or irresponsible consumers? Perceptions of professionals and patients

**DOI:** 10.1186/1756-0500-5-525

**Published:** 2012-09-25

**Authors:** Anne-Claire Durand, Sylvie Palazzolo, Nicolas Tanti-Hardouin, Patrick Gerbeaux, Roland Sambuc, Stéphanie Gentile

**Affiliations:** 1 Laboratoire de Santé Publique, Faculté de Médecine, Equipe de recherche EA 3279 “Evaluation hospitalière-Mesure de la santé perçue”, 27 boulevard Jean Moulin, 13385, Marseille cedex 5, France; 2 Service d’Accueil des Urgences, Hôpital de La Conception, 147 Boulevard Baille, 13385, Marseille cedex 5, France

**Keywords:** Emergency department, Health services needs and demand, Attitude of nonurgent patients, Qualitative study, Social perceptions of health professionals

## Abstract

**Background:**

For several decades, overcrowding in emergency departments (EDs) has been intensifying due to the increased number of patients seeking care in EDs. Demand growth is partly due to misuse of EDs by patients who seek care for nonurgent problems. This study explores the reasons why people with nonurgent complaints choose to come to EDs, and how ED health professionals perceive the phenomenon of “nonurgency”.

**Results:**

Semi-structured interviews were conducted in 10 EDs with 87 nonurgent patients and 34 health professionals. Interviews of patients revealed three themes: (1) fulfilled health care needs, (2) barriers to primary care providers (PCPs), and (3) convenience. Patients chose EDs as discerning health consumers: they preferred EDs because they had difficulties obtaining a rapid appointment. Access to technical facilities in EDs spares the patient from being overwhelmed with appointments with various specialists. Four themes were identified from the interviews of health professionals: (1) the problem of defining a nonurgent visit, (2) explanations for patients’ use of EDs for nonurgent complaints, (3) consequences of nonurgent visits, and (4) solutions to counter this tendency.

**Conclusions:**

Studies on the underlying reasons patients opt for the ED, as well as on their decision-making process, are lacking. The present study highlighted discrepancies between the perceptions of ED patients and those of health professionals, with a special focus on patient behaviour. To explain the use of ED, health professionals based themselves on the acuity and urgency of medical problems, while patients focused on rational reasons to initiate care in the ED (accessibility to health care resources, and the context in which the medical problem occurred). In spite of some limitations due to the slightly outdated nature of our data, as well as the difficulty of categorizing nonurgent situations, our findings show the importance of conducting a detailed analysis of the demand for health care. Understanding it is crucial, as it is the main determining factor in the utilization of health care resources, and provides promising insights into the phenomenon of ED usage increase. For reforms to be successful, the process of decision-making for unscheduled patients will have to be thoroughly investigated.

## Background

Over the past three decades, overcrowding in emergency departments (EDs) has become a serious problem for EDs in several developed countries [1-5]. This problem has been extensively described in the emergency medicine literature [1-7]. The most important factor contributing to ED overcrowding has been inadequate inpatient bed availability [8]. Indeed, patients who present to EDs often face long waiting times to be treated and, for those who require admission, even longer waits for inpatient hospital beds [9]. This problem was defined by the Australasian College for Emergency Medicine (ACEM) and labelled “hospital access block” [10]. It has also been described as an “international symptom of health care system failure” [11].

Additionally, ED overcrowding is amplified by the increased number of patients seeking care in EDs. In France, as in many other countries, ED visits increased by 4.3% every year between 1996 and 2008 [1,5,8]. Demand growth is partly due to inaccessibility to primary care services, and misuse of EDs by patients who seek care for nonurgent problems (i.e. problems which are not life or limb threatening or which do not require immediate attention) [1,12-14]. An extensive, recent international literature review reported that between 4.8% to 90% of ED patients were potentially nonurgent cases, with a median of 32.1% [12]. This review showed the lack of reliability and reproducibility of methods and criteria for categorizing ED visits into nonurgent cases, and that there is no easy way of determining the true burden of nonurgent patients in EDs [12]. The inaccurate estimate of nonurgent patients in EDs is of significant concern, as it leads to misguided strategies to reduce ED overcrowding that may be doomed to fail [15].

Many authors have researched why individuals choose to come to EDs instead of their primary care providers (PCPs) with nonurgent complaints [16-19]. Most of these studies are based on administrative data. Several preventive measures have been developed, including patient education programs on nonurgent ED use, alternative health care structures (named primary care units in France, called general practitioner (GP) consultations that do not require appointments or after-hours general practices in proximity to EDs in Anglo-Saxon countries), and after-hours telephone consultations [15,20]. Such strategies are often well funded and widely promoted as a solution to ED overcrowding [15].

However, few studies have focused on the congruence between the experience and perceptions of patients and those of ED health professionals to better understand the fundamentals of patients’ decision-making [21,22], and to discover robust strategies that could be developed to effectively mitigate ED overcrowding. Thus, the purpose of the current study was to produce an exploratory analysis of the decision-making process to seek care in the ED for a nonurgent illness. This was accomplished by evaluating the experience and perceptions of both patients and ED health professionals in the context of an ED visit. The study also aimed to explore how ED health professionals perceive the phenomenon of nonurgent ED patients, and to examine the solutions proposed by these professionals.

## Methods

### Research design

Using a qualitative descriptive design, we examined the problem of nonurgent ED visits through the experience and perceptions of patients visiting EDs and that of ED health professionals. We sought to provide a comprehensive summary of events in common, everyday language [23] that would reveal more in-depth and rich information than quantitative description. Overall themes and patterns were assessed using inductive content analysis [24].

### Setting

In March 2006, the study was conducted in 10 EDs located in the Provence-Alpes-Côte d’Azur (PACA) region of France. In 2006, the number of patient visits to these EDs ranged from 11,000 to 65,000.

### Study participants

To explore the problem of nonurgent ED visits, two categories of participants were interviewed:

The first sample consisted of patients who sought treatment in an ED. Study participants were patients aged 18 years or older who met the following eligibility criteria: they arrived by their own means, and were triaged as nonurgent upon their arrival to the ED by a triage nurse. To achieve a representative sample, patients were enrolled during one week, from 9 a.m. to 8 p.m. Each day, 2 time slots of 2 hours corresponding to peak ED consultation were randomly selected. Patients who required immediate medical care, had communication difficulties, or who were unable or unwilling to participate in the informed consent process were excluded from participating.

The second sample consisted of ED health professionals (physicians and nurses). Inclusion criteria were employment by one of 10 EDs selected, presence in the ED on the day of data collection, at least 6 months of professional ED experience, and willingness to participate.

#### Categorization of the urgency of the ED visit

According to an extensive literature review [12], there is no specific universal definition of a nonurgent ED visit. Most often, patients categorized as nonurgent are defined as those “who could have been treated by a general practitioner” [12,25].

In our study, categorization was conducted by triage nurses, immediately following the patient encounter. The categorization into urgent or nonurgent cases was done from a brief interview of the patient about their chief complaint and the history of that complaint.

Triage nurses were asked the rhetorical question, “According to the brief interview you just had with the patient, could this problem be taken care of by a primary care physician?” and, if the answer was yes, the ED visit was categorized as “nonurgent”.

Categorization was performed in a discrete manner without disturbing the activity of ED health professionals, i.e. without the use of written triage protocols or algorithms. All triage nurses who conducted the categorization were registered nurses and had a minimum of 6 months of experience in the care of ED patients. Triage nurses had not attended training sessions specifically for this study; however, categorization of urgency is part of their professional training [26].

### Data collection and procedure

Study data were collected in March 2006 by two research assistants with experience in qualitative research (AC.D. and S.P.). Following informed consent, researchers conducted in-depth interviews with study participants who met inclusion criteria and agreed to participate. Each interview lasted between 30 to 60 minutes in a private setting in the ED, and was recorded using a digital audio system. Researchers met to discuss their findings on an ongoing basis.

To explore the problem of nonurgent ED visits, two semi-structured interview guidelines were developed by two researchers (AC.D. and S.G.) based on their extensive review of the literature related to the problem of nonurgent ED visits, and methods and criteria for categorizing ED visits into nonurgent cases [12]. One guide addressed nonurgent ED patients and the other was for ED health professionals. The two semi-structured interview guidelines were pre-tested and validated by the study’s steering committee. Interview questions are listed in Table 1.

**Table 1:** Questions from interview guide

**Participants**	**Primary questions**
**ED patients**	1. Would you describe to me what happened to you today?
	2. Why did you choose to come to the emergency department today?
	3. Do you have access to an alternative source of care to treat your current problem?
	4. What do you usually do when you are sick?
	5. Do you think that the emergency department is the most appropriate place to treat your current health care problem?
	**Primary questions**
**ED health professionals**	1. Would you describe the patients who present to the emergency department for nonurgent complaints?
	2. In your opinion, why do nonurgent patients choose to come to the emergency department rather than their family practitioner?
	3. What are the consequences of such nonurgent visits?
	4. Do you have any solutions to limit nonurgent ED visits?

Specifically, the interview guide for nonurgent ED patients focused on the overall management of their health, their perceptions of what constitutes an urgent ED visit, and the decision-making process which led them to the ED at the time of the interview. For ED health professionals, the interview guide focused on their perceptions of what constitutes an urgent/nonurgent ED visit, causes and consequences of the phenomenon of nonurgent ED visits, and solutions to limit nonurgent ED visits.

At the end of the interview, a short questionnaire was administered to each participant. For both populations, the questionnaire dealt with socio-demographic data. The patient’s questionnaire included an additional question on their usual source of care, i.e. followed by a GP and health insurance status. The ED health professionals’ questionnaire included an additional question on employment status (profession, experience in EDs).

Participant selection, data collection until theoretical saturation was reached, and a rich description of experience and perceptions were obtained. This means that when new interviews no longer yielded new information and all potential sources of variation were adequately explored, sampling could stop [24,27].

### Data analysis

All interviews were tape-recorded and transcribed verbatim. Conventional content analysis was used to analyze the data [28]. The interviews were analyzed and interpreted by a multi-professional research team with different backgrounds: one sociologist (S.P.) with experience in injury and qualitative research, one ED physician (P.G.) with expertise in injury research, one public health physician (S.G.) with expertise in public health and qualitative research, and one research assistant and also PhD student (AC.D.) with expertise in public health and qualitative research. The researchers independently read and reread the transcripts. To ensure credibility of the data interpretations, the content analysis was conducted independently by the researchers. Each researcher then identified meaningful units/statements in the text, highlighted them, and transferred them to individual file cards [24]. The file cards were then categorized by question, and the researcher’s subjective interpretations of the meaningful statements were noted on each card. Similar statements and interpretations were then clustered. Following their individual activities, the researchers met to reach a consensus regarding the meaningful statements in the text as well as potential subjective interpretations of these statements. Whenever divergent interpretations occurred, transcripts were re-reviewed and discussed until consensus was achieved.

The cards were then examined for possible themes/categories. An additional check was conducted by an independent expert researcher who validated the themes.

### Ethical considerations

All participation was voluntary and confidential. Verbal consent was obtained and all participants were informed of the conditions of the study and that they could refuse to participate or withdraw from the interviews at any time. Our study was non-interventional, and did not need to be approved by an ethics committee under the criteria of the bioethics law [29]. Therefore, our study did not require authorization of the National Commission for Informatics and Freedom due to respect for patient anonymity [29].

## Results

Eighty-seven ED patients were categorized as nonurgent by a triage nurse, and 34 ED health professionals agreed to participate in the study.

### Nonurgent patients and their reasons for ED use

Of the 87 nonurgent ED patients interviewed, slightly over half were male (54.6 %). The mean age [± standard deviation (SD)] was 38.3 years ± 16.2 (range of 17 to 78 years). Most were employed (67.8 %), and all had primary health insurance with supplementary coverage; 9% of them were covered by French national health insurance, called “CMU,” for individuals and families with low income and resources. The majority of patient participants were followed by a PCP (82.8%).

During the ED visit, 76% of patients indicated that symptoms associated with their chief complaint had been present for less than 24 hours. Nearly one-third (32.1%) indicated they had tried to reach their PCP prior to coming to the ED. Most of the nonurgent ED patients interviewed were self-referred (79.3%); others were referred by a PCP (16.1%) or referred for medico-legal reasons (4.6%) (employer or police). Nearly half were consulting for minor traumatic problems (48.3%).

Three recurrent themes emerged from the patients’ interviews: nonurgent patients seek care in the ED because it fulfills health care needs, because of barriers by PCPs, and for the advantages of the ED setting (see Table 2).

**Table 2:** Reasons for ED use for nonurgent patients: category descriptions

**Theme and sub-category**	**Descriptors**
**Theme 1. Fulfill health care needs**	
To alleviate pain or discomfort	▪ “It’s urgent because it hurts”
	▪ “I suffered for a while there. I’ve been trying to tough it out, but I suffer too much.”
Anxiety generated by the complaint	▪ “I don’t consider my problem serious, but I am worried because I am hurting.”
	▪ “I do not know what I have, but it worried me, so I preferred to come immediately to the ED so at least I am reassured.”
	▪ “I was afraid; I was concerned because I did not know if my problem was serious.”
**Theme 2. Barriers to primary care providers**	
Difficulty obtaining an appointment with their PCP in a timely manner	▪ “When I called my doctor, he said that he was booked up, and he instructed me to go to the ED.”
	▪ “It is impossible to see him during the week if you are sick (speaking of her PCP). It is too long.”
	▪ “I called my doctor but he could not see me, so I preferred to come to the ED because the pain was unbearable.”
ED is the only alternative to accommodate work schedules	▪ “When I am sick and miss a day of work, I need to see a doctor that day. I can’t afford to be off work any longer. I need to feel better and go back to work the next day”
	▪ “After 6 p.m., nothing else is open.”
Discerning health consumers	▪ “I preferred the ED to my doctor because it is so hard to get in to see him.”
	▪ “I knew that my doctor could not see me. So, I came to the ED.”
	▪ “My doctor consults by appointment only. He doesn’t have time for me.”
**Theme 3. Advantages of the ED**	
Availability of diagnostic tests and treatment	▪ “My doctor cannot do X-rays or laboratory tests, while the ED has all the technical support.”
	▪ “I'd rather be here than run around. At least here x-rays can be done.”
Convenience	▪ “Everything is in one place.”
	▪ “The doctors perform things a lot faster.”

#### Fulfill health care needs

The first theme was “fulfill health care needs.” Alleviating pain or discomfort (cited in 35.6% of cases), and anxiety generated by the complaint (cited in 29.9% of cases) were the main needs contributing to the decision to use the ED. Pain was also considered as an emergency, as stated by several patients (see Table 2).

Reducing anxiety and being reassured were the major expectations of nonurgent ED patients, even if they knew their problem was not life-threatening (17.2% felt their medical needs were serious).

#### Barriers to primary care providers

The second theme was “barriers to primary care providers.” When they were coping with distress and when making decisions to seek care by consulting their PCPS, they had difficulty obtaining a timely appointment. The patients in our study indicated that the delay in obtaining an appointment was a major reason to seek care in an ED. Moreover, patients working during regular business hours had problems obtaining an appointment with their regular PCP before or after their workday. Even if patients were able to make an appointment, many of them did not want to take a day off to visit their PCP.

Patients interviewed chose the ED as discerning health consumers. Indeed, patients knew the health care system; they were very well informed about the health care system and the primary care services available to them. Therefore, they were able to identify possible alternatives, and to translate their assessments into choices.

#### Advantages of the ED

The final theme was related to several advantages of care specific to the ED setting. Availability of resources, including laboratory tests and radiography, was one of the advantages of the ED. These facilities were not available in PCP offices. Moreover, patients’ decisions were influenced by the relative convenience of being seen in the ED as compared to the PCP’s office. The access to technical facilities, the opportunity to be cared for in a single place, and the availability of medication were attractive ED attributes for many patients. These advantages spare patients the complexities of making several appointments in different places, and from being overwhelmed with organizational concerns. This is the case for patients with minor trauma complaints, which may require x-ray examination and/or surgical intervention.

Moreover, the need for ED facilities was related to the need for reassurance, as mentioned above. Several patients stated that they came to the ED to be reassured by x-ray tests and possibly a CT-scan.

### ED health professionals and their perceptions of “nonurgency”

Of the 34 ED health professionals interviewed, 73.5% were ED physicians (n = 25) and 26.5% were ED nurses (n = 9).

In the group of ED physicians, the mean age (± SD) was 41.9 years ± 8.9, and 64.0% were male. The majority (92%) had at least five years of experience in the ED. In the group of ED nurses, mean age (± SD) was 41.0 years ± 7.2, and one-third were female (33.3%). More than 75% had more than five years of experience in ED. When comparing the two sub-groups, no significant difference regarding the themes were found between them. Therefore, the results of both sub-groups have been aggregated.

Four main themes emerged from ED health professionals’ interviews: definition of a nonurgent visit and an inappropriate visit, the reasons for using EDs for nonurgent complaints, the consequences of nonurgent ED visits, and the solutions to counter this problem (see Table 3).

**Table 3:** Perceptions of ED health professionals regarding nonurgent ED patients

**Theme and sub-category**	**Descriptors**
**Theme 1. Problem of defining a nonurgent visit and an inappropriate visit**	
No specific definition	▪ “It’s easy to consider a nonurgent case at the end of the consultation, but it's very difficult in the triage area.”
Perception of what constitutes a nonurgent case	▪ “Anything that is not life-threatening.”
	▪ “A condition is nonurgent if it can be treated in 2 to 3 days.”
	▪ “Consultations are nonurgent if the chief complaint is a non-serious illness that can be treated by a PCP.”
Difference between nonurgent cases and inappropriate cases	▪ “If no other sources of care are available, patients have no other choice but to go to the ED. In this case, a nonurgent consultation could be considered appropriate.”
	▪ “All patients whose care can be given at a facility other than the ED.” ▪ “We must redefine what is an emergency, what is an appropriate visit to the
	ED, and what is inappropriate, but it is very difficult to define.”
**Theme 2. Reasons for using EDs for nonurgent complaints**	
Lack of access to PCPs	▪ “PCPs are not available evenings and weekends…”
	▪ “Continuity of care in primary care services is not guaranteed on Saturdays and Sundays.”
	▪ “In some geographical sectors, there is virtually no primary health care structure ensuring continuity of care. EDs are the only medical places available 24 hours a day, seven days a week.”
Health care consumerism	▪ “The use of care is similar to that of products, i.e. fast and easy… We are in the Internet age, where everything is readily available, and the use of health care is no exception to this trend.”
	▪ “The population evolves towards the need for rapid response to a need.”
	▪ “People want to receive care on the same day, including access to technical facilities.”
	▪ “Frustration is not acceptable”.
No advance payment at the time of the ED visit	▪ “Some patients come to EDs for financial reasons. There is a perception that the hospital is free, but it is not.”
	▪ “People believe that the medical consultation is free at the time of the ED visit, but the consultation is supported by our health insurance system.”
**Theme 3. Consequences of the increase in nonurgent ED visits**	
	▪ “It is a problem when there are peaks of activity…This increase in utilization of EDs has induced overcrowding, prolonged wait times, delayed diagnosis and treatments, reduced quality of care, and increased the risk of adverse outcomes.”
	▪ “Most ED colleagues are stressed because EDs are not structured for primary care.”
	▪ “They (ED colleagues) feel that they no longer practice emergency medicine.”
**Theme 4. Solutions to reduce the number of ED visits for nonurgent complaints**	
Patient education	▪ “We should communicate more about what is a real urgent problem.”
	▪ “Perhaps if people were educated regarding the importance of primary care and the appropriate use of EDs, they might seek ED care less often.”
To reorganize the health care system by improving the continuity of care outside regular business hours	▪ “We could have primary care consultations in close proximity to the ED. These consultations would be opened between 8 a.m. and midnight. When there is a real emergency, patients would be sent back to the ED.”
	▪ “A working collaboration between EDs and PCPs would improve accessibility to ensure that services are used effectively and efficiently.”
To integrate a “gatekeeper” at the ED	“To determine patients having authorization for care in ED, a physician should discern whether the consultation is appropriate or not.”
A financial penalty for patients categorized as nonurgent after the consultation	▪ “If it’s not urgent, we look after you, but you will pay - you will pay at least an “emergency fee”;
	▪ “No significant financial penalties to prevent the use of EDs exist.”

#### Problem of definition: nonurgent visit and/or an inappropriate visit?

The first theme was the “problem of defining a nonurgent visit and an inappropriate visit.” ED health professionals considered it essential to clarify the concept of a nonurgent visit because it is poorly defined. They often defined a nonurgent visit according to medical criteria, such as calling it a “minor medical problem that is non-acute, non life-threatening.” For them, the concept of “nonurgency” was in opposition to the concept of “vital urgency”; it is a problem that is not likely life-threatening, does not require immediate attention, and is considered as nonurgent because care can be delayed for several hours or days. Moreover, for ED health professionals, there was a distinction between “nonurgent” cases and “inappropriate” cases. Indeed, the term “nonurgent” indicates mainly the level of severity of the medical problem such as vital signs. In contrast, the term “inappropriate” covers, in addition to the medical problem, the social and psychological condition of patients, visiting hours (during business hours or not), and availability of heath care in the ED.

#### Reasons for using EDs for nonurgent complaints

The second theme was the “reasons for using EDs for nonurgent complaints.” More than half of the ED health professionals believed that lack of access to PCPs influenced patients to seek care in EDs. For them, patients were sometimes unsuccessful in gaining access to PCP offices. A major problem for patients was the long wait for appointments, and the lack of availability of PCPs on evenings and weekends.

Furthermore, all ED health professionals condemned the behavior of nonurgent patients who seek care in EDs. They thought that patients visiting the ED for nonurgent complaints wanted to decide how and when they should undergo treatment. Indeed, most of them described nonurgent patients as “abusive healthcare consumers.” They believed that the consumer mentality has spread to medicine, so that patients consume medical resources like any other resources. ED health professionals felt that patients who want everything immediately and indiscriminately are irresponsible.

In addition, most health professionals thought the financial dimension of the ED consultation contributed to the decision to use the ED. Professionals sharing this opinion further elaborated that patients do not realize the cost of care because they do not pay at the time of the consultation. Since patients are treated first and receive the bill later if their insurance does not fully cover the fees, they initially are often not aware of the magnitude of the costs for the health care system.

#### Consequences of the increase in nonurgent ED visits

ED health professionals considered that nonurgent patients decreased ED access for real emergency cases, reduced the quality of care (prolonged waiting times, delayed diagnoses and treatments, delayed care for seriously ill patients), and produced negative spillover effects. Moreover, nonurgent ED visits caused disproportionate frustration among staff, because ED health professionals had the impression that they were no longer practicing the kind of medicine that they trained for.

#### Solutions to reduce the number of ED visits for nonurgent complaints

To decrease the number of nonurgent ED visits, ED professionals proposed several solutions. The first solution was patient education regarding appropriate use of health care services so as to help them make more rational decisions. The second proposal (by 55.8% of professionals) was to reorganize the health care system by improving the continuity of care outside regular business hours. The third proposal was to integrate a “gatekeeper” in the ED who would require authorization from patients’ PCPs for admission to the ED. Finally, some suggested imposing a financial penalty for patients categorized as nonurgent after the consultation.

## Discussion

Our study revealed a discrepancy between the perceptions of ED health professionals and those of ED patients. Perceptions of ED health professionals were mainly based on the acuity and urgency of patients’ medical problems. Perceptions of ED patients, on the other hand, were based on medical factors, feelings (pain, anxiety), accessibility to health care resources, and practical concerns surrounding the medical problem (whether it took place at the workplace or at school, and whether it occurred during regular business hours). For patients who sought care in the ED for nonurgent medical problems, decision-making was most often linked with health care needs, as in previous studies [16,21]. In our study, patients found relief and reassurance in the ED setting. Beyond medical needs, the second most important reason was the availability of health care resources. Patients behaved as “rational consumers” [30,31] when choosing to go to the ED. Indeed, in our study, patients were fully informed about all alternative health care structures, treatments and services (doctors, insurance). Interviews showed that patients are able to use this information to make their own choice when selecting care providers. For them, among all health care resources available, the ED is the most suitable place and the most efficient provider that can fulfill their medical needs. EDs can deliver a full range of medical services, regardless of the presenting complaint, and it is accessible 24 hours a day, 7 days a week [14]. These numerous advantages do not exist in PCP offices where appointment availability can be sparse, and opening hours restricted. This may be an important factor for patients who work during business hours, and who may therefore have difficulty going to a PCP.

The notion of “rational consumer” was not at all acknowledged by the interviewed health professionals, who stigmatized patients as “abusive and irresponsible consumers” of health care resources. For them, patients use EDs to be seen earlier and faster than their medical condition warrants. ED professionals argued that their first medical mission is specifically to care for life-threatening problems. Therefore, pain, physical discomfort, and even minor traumatic complaints were often viewed by health professionals as unjustified ED use. Regarding this point, ED health professionals and patients differed considerably in what they consider an urgent case and an appropriate ED complaint.

ED patients were willing to optimize their medical care by considering the most efficient health care resources. In their eyes, the ED seems to be the most appropriate health care resource for acute complaints, which require prompt care. Therefore, the solutions proposed by ED health professionals to reduce the number of nonurgent ED patients are irrelevant. This is because they are based on misconceptions about the behavior and attitudes of nonurgent patients, as evidenced by studies reporting educational interventions which target nonurgent patients [20].

In addition, the other solutions proposed by health professionals on limiting access to EDs (financial penalties and/or the preauthorization) could be applied if the PCPs availability for treatment of minor trauma were adequate. The methods of triage must guarantee the safety of the patients.

Finally, the last solution proposed was to set up PCP structures in proximity to EDs. Patients could access them after ED triage or directly. This solution would, in theory, be the best. In a previous study, our research team assessed the willingness of nonurgent patients to be referred outside the ED to a PCP [32]. Patients categorized as nonurgent in EDs were willing to use PCPs if they were in close proximity, if the time slots between EDs and PCPs were compatible, and if minor surgery could be performed there. Therefore, this solution appears to receive support from patients. If the funding were available, this initiative may indeed help ED crowding.

### Limitations

Interpretation of this study is somewhat limited. Our analysis does not distinguish perceptions of nurses from those of physicians. However, there is no significant difference in terms of perceptions between the two sub-groups. Another issue is that our data dates back to 6 years ago. Nevertheless, information from ED healthcare professionals and patients remains valid and relevant. There is a climate of increased demand for healthcare along with a lack of definition of nonurgent or inappropriate ED visits. Indeed, what constitutes the nonurgent or inappropriate ED patient is still debated in the literature both nationally and internationally [12,33]. Understanding patient motivations for seeking healthcare may assist in the development of demand management strategies.

## Conclusion

Subject to these limitations, our study highlighted discrepancies between ED patients and ED health professionals, especially regarding patient behavior. Given the lack of thorough analysis of the health care demand for acute complaints, which do not involve life-threatening situations, health professionals’ proposals to reduce the number of nonurgent visits are doomed to fail. Our findings emphasize the importance of conducting a detailed analysis of the demand for health care, especially due to the fact that it is the main determining factor for utilization of health care resources, and a pertinent explanation for the increased use of EDs. For reforms to be successful, the process of decision-making of unscheduled patients, including those with minor trauma, will have to be thoroughly investigated.

## Competing interests

The authors declare that they have no competing interests.

## Authors’ contributions

ACD and SG participated in the design and the coordination of the study, performed the statistical analysis and helped to draft the manuscript. SG, ACD, SP, NTH and PG participated in the design of the study, interpreted the results and helped to draft the manuscript. RS participated in the statistical analysis and helped to draft the results. All authors read and approved the final manuscript.
